# Infectious diseases in allogeneic haematopoietic stem cell transplantation: prevention and prophylaxis strategy guidelines 2016

**DOI:** 10.1007/s00277-016-2711-1

**Published:** 2016-06-24

**Authors:** Andrew J. Ullmann, Martin Schmidt-Hieber, Hartmut Bertz, Werner J. Heinz, Michael Kiehl, William Krüger, Sabine Mousset, Stefan Neuburger, Silke Neumann, Olaf Penack, Gerda Silling, Jörg Janne Vehreschild, Hermann Einsele, Georg Maschmeyer, on behalf of the Infectious Diseases Working Party of the German Society for Hematology and Medical Oncology (AGIHO/DGHO) and the DAG-KBT (German Working Group for Blood and Marrow Transplantation)

**Affiliations:** 1 Department of Internal Medicine II, Division of Hematology and Oncology, Division of Infectious Diseases, Universitätsklinikum, Julius Maximilian’s University, Oberdürrbacher Str. 6, 97080 Würzburg, Germany; 2 Clinic for Hematology, Oncology und Tumor Immunology, Helios Clinic Berlin-Buch, Berlin, Germany; 3 Department of Hematology/Oncology, University of Freiburg Medical Center, 79106 Freiburg, Germany; 4 Medical Clinic I, Klinikum Frankfurt (Oder), Frankfurt (Oder), Germany; 5 Haematology and Oncology, Stem Cell Transplantation, Palliative Care, University Hospital Greifswald, Greifswald, Germany; 6 Medizinische Klinik III, Palliativmedizin und interdisziplinäre Onkologie, St. Josefs-Hospital Wiesbaden, Wiesbaden, Germany; 7 Sindelfingen-Böblingen Clinical Centre, Medical Department I, Division of Hematology and Oncology, Klinikverbund Südwest, Sindelfingen, Germany; 8 Medical Oncology, AMO MVZ, Wolfsburg, Germany; 9 Hematology, Oncology and Tumorimmunology, Charité University Medicine Berlin, Campus Virchow Klinikum, Berlin, Germany; 10 Department of Internal Medicine IV, University Hospital RWTH Aachen, Aachen, Germany; 11 Department I of Internal Medicine, German Centre for Infection Research, Partner-site: Bonn-Cologne, University Hospital of Cologne, Cologne, Germany; 12 Department of Hematology, Oncology and Palliative Care, Klinikum Ernst von Bergmann, Potsdam, Germany

**Keywords:** Infections, Viral, Fungal, Bacteria

## Abstract

Infectious complications after allogeneic haematopoietic stem cell transplantation (allo-HCT) remain a clinical challenge. This is a guideline provided by the AGIHO (Infectious Diseases Working Group) of the DGHO (German Society for Hematology and Medical Oncology). A core group of experts prepared a preliminary guideline, which was discussed, reviewed, and approved by the entire working group. The guideline provides clinical recommendations for the preventive management including prophylactic treatment of viral, bacterial, parasitic, and fungal diseases. The guideline focuses on antimicrobial agents but includes recommendations on the use of vaccinations. This is the updated version of the AGHIO guideline in the field of allogeneic haematopoietic stem cell transplantation utilizing methods according to evidence-based medicine criteria.

## Introduction

Infectious complications remain a clinical challenge in the setting of allogeneic haematopoietic stem cell transplantation (allo-HCT). Particular during the early phase after allo-HCT, mortality rates for infections are high [1, 2]. After the first publication of recommendations from our group in 2003, [3] numerous new results of trials have been published and implemented into daily patient care. With this updated guideline, AGIHO (Infectious Diseases Working Group) of the DGHO (German Society for Hematology and Medical Oncology) pursues a step forward to include the entire patient history up right from the beginning of the preparation of patients through the entire post allo-HCT time period.

This guideline focuses on the adult patient population only and is partitioned into four parts: (a) general precautions and prevention measures, (b) pre-transplantation (screening) phase, (c) prophylactic treatment, and (d) immunization strategies.

## Methods

Several steps were undertaken to develop the updated guideline: The first step was defining a group of specialists. They were enlisted by the AGIHO of the DGHO with a designated coordinator. The coordinator was responsible to manage the efforts of the group. The group of authors consisted of 14 certified internists, including 13 certified haematologists, and 5 certified infectious diseases specialists. Four authors are triple certified in internal medicine, infectious diseases, and haematology/oncology.

Predefined topics were elaborated by subgroups and then presented to the entire group for discussions. This included several face-to-face meetings, which were complemented by conference calls. Once the group had consensus with their results, the preliminary recommendations of the group were presented to the entire AGIHO assembly for review, discussions, modification, and final approval. All recommendations were made on the basis of available data providing evidence-based medicine. The guideline utilized the latest version of the strength of recommendation and quality of evidence published by the ESCMID (Table 1) [4]. Specific topics related to cord blood or haplo-identical transplant recipients are not addressed by this guideline.

**Table 1:** Strength of the AGIHO (DGHO) and DAG-KBT recommendation and quality of evidence (modified according to [4])

Strength of a recommendation	
Grade A	AGIHO strongly supports a recommendation for use
Grade B	AGIHO moderately supports a recommendation for use
Grade C	AGIHO marginally supports a recommendation for use
Grade D	AGIHO supports a recommendation against use
Quality of evidence	
Level I	Evidence from at least 1 properly designed randomized, controlled trial
Level II^a^	Evidence from at least 1 well-designed clinical trial, without randomization; from cohort or case-controlled analytic studies (preferably from >1 centre); from multiple time series; or from dramatic results of uncontrolled experiments
Level III	Evidence from opinions of respected authorities, based on clinical experience, descriptive case studies

^a^Added index: _**r**_ **:** meta-analysis (or systematic review of RCT); _**t**_ **:** transferred evidence i.e., results from different patients ‘cohorts’ or similar immune status situation; _**h**_ **:** comparator group: historical control; _**u**_ **:** uncontrolled trials; _a_ **:** published abstract (presented at an international symposium or meeting)

## General precautions

An allo-HCT requires certain assessment procedures, which are basically standardized (e.g. JACIE by the EBMT). Herein, we touch off on a few basic standardized requirements.

Patients’ rooms should be equipped with air-filtered systems to keep spore counts low and, thus, preventing nosocomial fungal diseases **(BII**) [5–9]. Further, nearby construction activities should be kept to a minimum **(AII)**.[10] Isolation of the stem cell recipients in a single hospital room under conditions of laminar airflow or positive pressure HEPA filtration (>12 exchanges per hour) is generally recommended.

However, randomized controlled trials focusing on HEPA filter efficacy against viral infections are lacking. Especially respiratory virus outbreaks, including seasonal pathogens such as respiratory syncytial virus (RSV) and influenza, are not prevented by HEPA filtrations [11]. Genotyping of RSV outbreaks demonstrated that more than two thirds were hospital acquired [12–15]. These results underscored the important necessity of infection control measures (i.e. barrier precautions) to prevent exposure directly at the patients’ site **(AII)**.

Some debate usually arises on the topic of appropriate dietary needs for patients after allo-HCT. The rule of thumb “cook it, peal it, or forget it” is easy to understand. However, there is a lack of appropriate literature on this specific topic. On the other hand, the evidence is clearer for the prevention of specific infections, e.g. listeria or other agents causing infectious diarrhea **(BII)** [16, 17]. Contact precautions and hand disinfection (incl. repeated teaching on this matter) can prevent nosocomial infection **(AII**) [18]. Healthcare workers (HCW) with transmissible diseases (e.g. herpes, infectious gastroenteritis, respiratory tract infections) should be restrained from direct patient care to prevent any nosocomial spread of their disease **(AIII)** [19]. Some hospital facilities have recovered microbes (e.g. *Legionella* spp.) from their drinking water. In order to prevent transmission in high-risk patients, water filters provide a protective solution though regular testing remains a necessity **(AII)** [20–22].

## Pre-transplantation (screening) phase

A comprehensive pre-transplant assessment of the allo-HCT recipient for infectious complications is a valuable tool to identify patients at increased risk for distinct infectious diseases.

Syphilis, tuberculosis, *Toxoplasma gondii*, HIV, hepatitis B and C viruses, and *Herpes viridae* usually persist lifelong in the host after primary infection and can be reactivated under certain conditions. As a consequence, all candidates for allo-HCT should undergo a test for IgG antibodies specific for viral diseases, syphilis, and toxoplasmosis. False negative results particularly could occur in the context of CLL, multiple myeloma, previous antibody treatment (e.g. rituximab), or might be false positive after IVIG application or blood product transfusion. In any case, all patients tested IgG-seronegative strictly remain on preventive measures to avoid de novo infection prior to allo-HCT and afterwards.

### Specific viruses

#### *Herpes viridae*

All candidates for allo-HCT should be tested for CMV, EBV, and VZV IgG antibodies to determine their risk for reactivation or de novo infection **(AIII)** [23–25]. Due to the high prevalence of HSV in the patient population, further antibody testing for HSV is not mandatory **(CII**_**t**_**)** [26].

#### Hepatitis B

Prior to allo-HCT, besides hepatitis B virus (HBV) antibody panels, additional testing for hepatitis B surface antigen (HBsAg) should be performed [27, 28]. If tested positive for HBsAg or for anti-HBc, further HBV-DNA assessment for active replication is crucial **(AII)**. If considered to be diagnosed with active hepatitis (e.g. viral replication), initiation of antiviral treatment prior to allo-HCT should be considered **(AIII)** [29].

There is a reported risk of up to 50 % for reverse seroconversion after allo-HCT if a patient is anti-HBc positive but has no detectable viral replication (resolved HBV infection) [30–32]. HBV-vaccination after allo-HCT might alleviate this risk [33].

#### Hepatitis C

Serologic testing for hepatitis C virus (HCV) is recommended. Serologically positive patients should receive quantitative testing for HCV-RNA viral load (**AIII**). Patients with chronic hepatitis C should receive a further diagnostic assessment, e.g., fibroscan or a liver biopsy to rule out liver fibrosis or cirrhosis. In case of liver cirrhosis or fibrosis, the conditioning regimen should try to avoid TBI, oral busulfan, or cyclophosphamide to minimize risk of hepatic sinusoidal occlusion syndrome (SOS) **(BIII)** [34–37].

#### Hepatitis E

Hepatitis E virus (HEV) is detected in immunocompromised patients. Limited information is available on the real incidence of HEV infection in recipients of allo-HCT [38, 39]. Mostly self-limited reactivation cases are published though chronic forms have been described as well. Serologic testing for HEV prior to allo-HCT is recommended **(BIII)**. HEV should be considered as a differential diagnosis in patients after allo-HCT with elevated liver function tests [39–41].

#### HIV

HIV testing prior to allo-HCT is recommended. HIV-infected patients should be carefully evaluated for allo-HCT. Though HIV seropositivity per se is not a contraindication for allo-HCT [42]. If allo-HCT seems feasible, a donor screening for CCR5-Delta 32 deletion could be considered in patients with CCR5 tropism to potentially control HIV infection post-allo-HCT **(BIII)** [43, 44]. Toxicity permitting, antiretroviral therapy should be continued throughout of the post-transplantation phase **(AII)** [45]. However, recurring interruptions with low drug levels may induce viral resistance, and an interrupted treatment should not be reinstated until the patient has sufficiently recovered to allow stable tablet intake **(BIII)**.

#### Syphilis

Serologic testing for syphilis is recommended. Frequently TPHA/TPPA or VDRL are utilized. Important are the combinations of nontreponemal (e.g. VDRL) and treponemal tests. If a nontreponemal test is positive, confirmation of infection with treponemal test (e.g. TPPA or TP-EIA) should be performed. In case of an active infection or unclear whether the patient received an adequate treatment in the past, a treatment with penicillin should be instituted **(BIII)** [46].

#### Toxoplasmosis

All candidates for allo-HCT should undergo serologic testing for toxoplasmosis. If the serology testing for toxoplasmosis IgG is positive, patients have a risk of toxoplasmosis reactivation, especially if the donor is serologically negative for toxoplasmosis [47]. Some centres propagate regular PCR testing [48]. Since the incidence in Europe is very low, regular toxoplasmosis DNA through PCR screening is not recommended **(DIII)**. This is of course different in patients with clinical symptoms.

#### Tuberculosis

Thorough evaluation of the medical history can identify patients at risk for latent or active tuberculosis infection **(AIII)**. As most candidates have received chemotherapy or immunosuppressive treatment prior to evaluation for allo-HCT, a tuberculin skin test might be false negative and therefore cannot be recommended in this setting **(DIII)**. If the medical history is suggestive of prior tuberculosis exposure, an interferon-gamma-release assay (IGRA) can be considered **(BIII)** [49]. However, a reduced sensitivity in immunocompromised patients has been demonstrated as well [50, 51].

## Prophylaxis and prevention

### Prevention of bacterial infections (screening for bacterial colonization)

In this era of easy accessibility of antibiotics, clinicians are facing the growing challenge of multi-resistant bacteria (e.g. vancomycin-resistant *Enterococci* (VRE), methicillin-resistant *Staphylococcus aureus* (MRSA), extended-spectrum beta-lactamase producing bacteria (ESBL), metallo-*ß*-lactamase-producing bacteria (MBL)). Colonization with certain multi-resistant bacteria is predictive for developing bloodstream infection, and knowledge of colonization status may therefore guide empirical antibiotic treatment, although this strategy has not been demonstrated to improve outcomes [52, 53]. We recommend screening procedures for multi-resistant bacteria, especially in institutions with a known high prevalence **(BII)** [54, 55]. Since the sensitivity of the screening methods is low, repeated testing (e.g. weekly rectal swabs) would be required [56–58]. Contact precautions between medical staff and patients remain to be necessary and separate sanitary facilities need to be guaranteed to exclude cross-patient transfer of multi-resistant bacteria [59, 60].

Antibiotic prophylaxis (e.g. ciprofloxacin) demonstrated its efficacy by reducing the incidence of Gram-negative sepsis during neutropenia without any significant change in mortality [61, 62]. All-cause mortality was reduced only in meta-analysis [63]. At institutions with a low rate of multi-resistant gram-negative bacteria, antibacterial prophylaxis remains a reasonable choice to reduce the incidence of Gram-negative sepsis during neutropenia **(AII**_**r**_**)** [62, 64–67]. Encapsulated bacteria such as *Streptococcus pneumoniae* or *Haemophilus influenzae* often cause severe bacterial infections in the late phase after transplantation [68–70]. In patients with chronic GVHD (graft-versus-host disease) on immunosuppressive therapy, antibiotic prophylaxis against these bacteria for disease prevention might be useful until immunizations can be applied **(BIII)**. Antibiotic selection should be guided by local antibiotic resistance patterns. In patients without chronic GVHD, systemic antibiotic prophylaxis is not recommended after reconstitution of the neutrophils **(DIII)**.

The value of selective gut decontamination is frequently debated and the literature points out that sepsis rates are increased and mortality outcomes were significantly worse in patients with lower intestinal diversity; therefore, no recommendation was made [71–74].

### Prophylaxis against *Pneumocystis* pneumonia

*Pneumocystis jirovecii* (previously named *Pneumocystis carinii*) pneumonia has been noted in allo-HCT recipients with an incidence of approximately 5–16 % without adequate prophylaxis and occurred at a median of 9 weeks after allo-HCT. Despite intensive treatment, mortality rates are as high as 89 % during the first 6 months and approximately 40 % after the first 6 months following allo-HCT [75, 76].

Prophylaxis against *Pneumocystis jirovecii* pneumonia is recommended for at least first 6 months after allo-HCT to prevent *Pneumocystis jirovecii* pneumonia-associated death **(AII**_**t**_**)**. However, patients might require prophylaxis for prolonged periods of time. Recommended prophylactic regimens are similar to regimens in HIV/AIDS patients. Therefore, patients on immunosuppressive medications or active GVHD should remain on prophylaxis [77]. Once immunosuppressive medications are discontinued or no active GVHD is noted, prophylaxis may be discontinued assuming a CD4^+^/CD3^+^ lymphocyte count of 200/μL or higher **(BII**_**t**_**)**. Thus monitoring of CD4^+^/CD3^+^ lymphocytes could be continued until the threshold is confirmed by repeated testing **(BII**_**t**_**)** [78]. The CD4^+^/CD3^+^ lymphocyte count of 200/μL as a discontinuation criterion is not confirmed in the allogeneic setting, and therefore, an individual decision to discontinue can be considered **(CIII)**.

The prophylactic treatment of choice is the fixed combination of trimethoprim (80 mg) and sulfamethoxazole (400 mg) once daily thrice weekly (**AII**_**t**_) [79–82]. In case of intolerance to the trimethoprim/sulfamethoxazole therapy, aerosolized pentamidine (300 mg) every 4 weeks **(BII**_**t**_**)** or atovaquone (750 or 1500 mg daily) **(BII**_**t**_**)** is recommended [83–89]. Dapsone (100 mg) cannot be recommended **(DII**_**t**_**)** [90]. Protective efficacy against *Pneumocystis* appears to be less with these alternative drugs compared to trimethoprim/sulfamethoxazole [84, 86, 90–93].

### Antifungal prophylaxis in allo-HCT

Invasive fungal diseases (IFDs) are severe complications associated with prolonged hospital length of stay, costs, long-term treatment, and high mortality [94]. Approximately two thirds of the IFD develop in allo-HCT patients after leukocyte recovery [95, 96]. Furthermore, intensifying immunosuppression for treatment of transplant rejection or GvHD and CMV infection impose an imminent risk for IFD [97, 98].

The incidence of invasive aspergillosis (IA) varies between reports and may reach 23 % [94, 99]. Primary prophylaxis is highly recommended since diagnostic tools do not present with sufficient sensitivity numbers. This is mirrored in studies with a significant number of post-mortem diagnoses of fungal diseases [100–102]. In patients diagnosed with IA, mortality rates of up to 60 % have been reported despite adequate treatment [103]. Secondary prophylaxis is recommended prior to allo-HCT **(BII)** [104].

Invasive candidiasis, predominantly manifesting as candidemia, is the second most frequent IFD in allo-HCT patients. Invasive candidiasis/candidemia typically manifests in patients with underlying conditions after being exposed to additional risk factors, e.g. intravascular devices, broad-spectrum antibiotic treatment, total parenteral nutrition, or *Candida* colonization [105–107].

The currently largest cohort of IFDs shows an 8 % share of mucormycosis in all IFDs in allo-HCT, followed by a number of other rare mould infections [94]. Approximately half of the patients diagnosed with mucormycosis are patients after allo-HCT [108]. The share of rare IFDs like those caused by the order of *Mucorales* or *Fusarium* spp. appear to be increasing [94, 109]. Newer agents like isavuconazole demonstrated favorable response rates in primary treatment against moulds; however, larger prophylaxis studies are still needed [110–112].

In Table 2, prophylactic recommendations are summarized. Our group recently published recommendations for the treatment (i.e. targeted therapy) of fungal diseases [113].

**Table 2:** Antifungal prophylaxis

Intention	Intervention	SoR	QoE	Comments	Ref
Prevent mould infection in patients without GvHD, day 1–100	Voriconazole 200 mg bid oral or iv^b^	C	I	No difference seen in the trial in comparison to fluconazole	[114]
	Posaconazole (suspension) 200 mg tid^b^	B	II_t_	Improved overall survival in AML/MDS induction during neutropenia, new formulations (tablet and iv, 300 mg qid) provide a better bioavailability	[115–117]
	Micafungin 50 mg/day	C	I	Only during neutropenia, morbidity advantage	[118]
	Itraconazole suspension 2.5–7.5 mg/kg or capsules	C	I	Administered up to 180 days if GVHD was diagnosed; higher toxicity in comparison to fluconazole, TDM: cutoff at 500 mg/mL (AII)	[119–121]
Prevent invasive *Candida* disease in patients without GvHD, day 1–100	Fluconazole 400 mg/day	A	I	Improved survival, note rising incidence of resistant *Candida* species since studies were published	[122–124]
	Voriconazole 200 mg bid oral or iv^b^	B	II_t_	Also active against moulds, but no difference seen in the trial between voriconazole and fluconazole	[114]
	Posaconazole (suspension) 200 mg tid^b^	B	II_t_	Also effective against moulds, new formulations (tablet and iv, 300 mg qd) provide a better bioavailability	[115, 117]
	Micafungin 50 mg/day	B	II_t_	Also effective against moulds, only during neutropenia, morbidity advantage	[118]
	Itraconazole suspension 2.5–7.5 mg/kg or capsules^b^	C	I	See above	[119–121]
Prevent invasive Aspergillosis during GvHD	Posaconazole (suspension) 200 mg tid^b^	A	I	improved survival (lower attributable mortality), new formulations (tablet and iv, 300 mg qd) provide a better bioavailability	[117, 125]
Prevent fungal disease relapse (previous IFD)	Voriconazole^b^	B	II	considered as secondary antifungal prophylaxis, dosages as above	[126]
	Caspofungin, posaconazole	B	III		[127, 128]
Prevent fungal diseases^a^	Amphotericin B deoxycholate	D	II	Inacceptable toxicity	[129–131]

^a^other formulations and various dosages and application regimens of Amphotericin B have been evaluated with different results in small studies, all would need further evaluation to provide any kind of recommendation [132–134]

^b^Consider TDM, serum levels of efficacy in prophylaxis are still debated, e.g. posaconazole [135]

### Herpes simplex virus 1/2 prevention

*Herpes viridae* persist in the host after primary infection. Up to 80 % of adults are HSV-seropositive and especially during immunosuppression HSV may begin to replicate. Without prophylaxis allo-HCT recipients have a risk of approximately 80 % to reactivate during the early phase mainly during the first 4 weeks after allo-HCT [19, 137, 138]. Dissemination may lead to severe illness with substantial morbidity and mortality. As a consequence, patients should receive acyclovir early on for the prevention of disease to reduce mortality **(AI)** [138–141] (Table 3).

**Table 3:** Antiviral prophylaxis

Intention	Intervention	SoR	QoE	Comments	Ref
HSV					
Prevent HSV disease	Acyclovir 400 mg tid/day	A	II	Up to 30 days post allo-HCT (various dosages)	[139, 141, 233]
	Valacyclovir 500 mg bid /day	A	II		[234]
	Acyclovir any dosage	D	III	Beyond 30 days if patient is also VZV seronegative	
VZV					
VZV disease prevention in VZV seropositive recipients	Acyclovir 800 mg bid	A	I	Up to 1 year after allo-HCT	[156]
	Acyclovir 400 mg/day	B	II		[158, 159, 235]
	Valacyclovir 500 mg bid	B	II		[236, 237]
	Acyclovir 200 mg/day	B	II	More than 365 days if continued on immunosuppressive therapy	[233, 238]
Prevent VZV in seronegative patients	No prophylaxis	C	III		
Prevent VZV in seronegative patients if exposed	Passive immunization	C	II_t_	Within 96 h post exposure, optional	[155]
	Acyclovir or other VZV-active antiviral	C	III	If patient is not on acyclovir (or any other VZV active antiviral), a short duration of therapy is an option.	
Prevent VZV disease after exposure	Vaccination	–	–	No data to provide recommendation	
CMV					
Preemptive strategy recommended over prophylaxis/treatment	Ganciclovir, valganciclovir, or foscarnet	A	I		[177, 239–242]
Reduce incidence of CMV infection/disease, if a center does not follow a preemptive strategy	Long term acyclovir 800 mg/day	C	II		[180]
	Valacyclovir 500 mg qid/day	B	I		[182]
	Ganciclovir 2.5–5 mg/kg bid/day	C	II	Caution: myelotoxicity	[243, 244]
	Valganciclovir 900 mg bid	A	II	Caution: myelotoxicity	[177]
	CMV-specific CTLs	C	II	Not available at every site (considered experimental)	[245]
HBV					
Prevent disease in HBsAG seropositive recipients	Lamivudine 100 mg/day	A	II	Monitor HBV DNA closely, duration until anti-HBs is detected (and HBV-DNA is negative)	[210, 246, 247]
	Entecavir 0.5–1.0 mg/day	A	II		[248–250]
	Tenofovir 245 mg/day	C	III		[251, 252]
Prevent disease in HBsAG seropositive recipients with HBsAG seronegative donors	Additionally vaccinate donor	B	III	Requires long term planning	[253]
Prevent reactivation in recipients who are anti-HBcAG seropositive, DNA viral load: positive	Lamivudine 100 mg/day	B	III		[209]
Prevent reactivation and disease in recipients who are anti-HBcAG seropositive, DNA viral load: negative	Lamivudine 100 mg/day	C	III		
	HBV-DNA/HBsAG monitoring	B	III		[254]

The duration of prophylaxis should last for up to 30 days after allo-HCT **(AI)** [139, 141]. However, exceptions are defined by recurrent episodes of HSV disease or risk of *Varicella zoster* disease. In these situations, duration of acyclovir prophylaxis to prevent disease is prolonged to a year or longer especially during intensified immunosuppressive therapy **(BII)** [142].

Resistance to acyclovir is a rare event and mainly caused by reduced activity or mutations of viral thymidine kinase resulting in reduced activation of acyclovir in infected cells [143, 144]. Breakthrough infections are noted but are usually described as clinically resistant since prophylaxis failure is explainable by decreased bioavailability of acyclovir. In cases of real acyclovir-resistant HSV, foscarnet susceptibility remains, and this agent is considered as an alternative treatment option for acyclovir-resistant disease **(BII)**. However, it cannot be recommended for routine prophylaxis due to its significant toxicity **(DIII)** [145].

It is presumed that valacyclovir and famciclovir are effective for the prevention of HSV reactivation; however, there are no clinical trials in allo-HCT to better support a recommendation **(CIII)** [146, 147].

### *Varicella zoster* virus prevention

Since *Varicella zoster* virus (VZV) is highly contagious, patients with VZV disease should be isolated to prevent nosocomial spreading of viruses until all lesions are crusted **(AIII)** [148, 149]. Patients should be informed of the easy transmission of VZV. Allo-HCT recipients without adequate antiviral prophylaxis are at risk for disease, since up to two thirds develop herpes zoster, which mainly occurs 3 to 12 months after allo-HCT [150]. VZV seronegative family members, healthcare workers, other contact persons of allo-HCT recipients, or children without a history of *Varicella* or immunization, should be advised to receive a vaccination against VZV ideally at least 4 weeks prior to planned allo-HCT **(BIII)** [151].

Primary infection is rare and is associated with a high rate of mortality caused by frequent dissemination (e.g. encephalitis, pneumonia, viscera, or hepatitis) [150, 152, 153]. Therefore, exposure of seronegative recipients to chickenpox, zoster or vaccinated persons who experience a rash after vaccination should be avoided to prevent primary disease or VZV-associated death **(BIII)**. If exposure to persons with chickenpox or zoster occurs, passive immunization with anti-VZV hyperimmunoglobulin (Ig) within 96 h after exposure is considered optional **(CIII)**, as efficacy has not been proven [154, 155]. Antiviral therapy with valacyclovir 1 g po tid or acyclovir 800 mg qid should be administered **(BIII)** immediately to prevent disease in seronegative recipients.

Main antiviral prophylaxis recommendations are summarized in Table 3. Various authors [156–159] noted that even prolonged administration for approximately 1 year or longer is considered safe and there was no higher incidence of disease after drug discontinuation. Longer than 12-month periods appears to be beneficial as long as patients remain on intensified immunosuppressive therapy **(BIII)**.

If resistance to acyclovir is suspected, foscarnet or cidofovir are alternative agents **(BIII)** [160]. Brivudine is contraindicated in patients receiving 5-fluoropyrimidine derivatives and was not assessed in immunocompromised patients **(DIII)**. (http://www.bfarm.de/SharedDocs/Risikoinformationen/Pharmakovigilanz/EN/RHB/2012/rhb-zostex.html, last accessed May 1, 2016)

### CMV disease prevention

All CMV-seronegative recipients ideally should receive a CMV-seronegative donor graft to prevent infection and reduce mortality **(AII)** [25, 161, 162]. To further prevent disease, CMV-seronegative recipients transplanted from a negative donor should only receive blood products from CMV-seronegative donors upon availability. Blood banks without a sufficient pool of CMV-negative donors should deliver only leukocyte-depleted red blood cells and thrombocytes **(AII)** [163]. However, data from various studies suggest if blood products are leukocytes reduced, testing for CMV-negative blood products is not needed for HSCT recipients **(AII)** [163–165]. Noteworthy, irradiation to prevent transfusion-associated GvHD does not inactivate CMV [164, 166–170].

Special risk factors for CMV infection or disease are T cell-depleted graft, HLA-mismatched transplantation, steroid treatment, and acute or chronic GvHD [171–173]. All patients at risk for CMV disease should be screened regularly for pp65 antigenemia or by nucleic acid detection methods after allo-HCT **(AII)** [174].

The current standard to improve morbidity and lower mortality is the early initiation of a preemptive therapy against CMV **(AII)** [174]. Duration of screening is usually defined by the time period of the application of immunosuppressive agents or GVHD.

Anti-CMV prophylaxis can only be considered as an option. The long-term administration of ganciclovir resulted in a delay of recovery from CMV-specific T cell immunity [175]. Valganciclovir is so far not officially approved in allo-HCT patients but has been applied in randomized trials [176, 177]. In a randomized controlled trial, valganciclovir prophylaxis was not superior in reducing the incidence of CMV disease or death when compared with PCR-guided preemptive therapy. Delay in virus-specific T cell reconstitution was not observed in patients receiving prophylaxis [177]. Administration of human immune globulins for prophylaxis or therapy of CMV disease is generally not recommended **(DII)** [178, 179]. Some investigators published efficacy of high-dose acyclovir or its prodrug valacyclovir in the prevention of CMV disease [180–183]. However, acyclovir failed to prevent CMV disease in autologous transplantation and therefore, is not recommended for prophylaxis **(DIII)** [184].

Newer antiviral agents have been evaluated mainly in phase II trials. Maribavir, an oral antiviral agent was studied for prophylaxis. Maribavir inhibits the UL97 viral protein-kinase of human CMV. Despite promising results in a phase II study, a phase III study could not confirm a benefit [185–187]. Another antiviral agent named CMX001 is an orally bioavailable lipid acyclic nucleoside phosphonate and is converted intracellularly to cidofovir diphosphate. Brincidofovir (CMX001) is active in vitro against CMV, including ganciclovir-resistant strains and was assessed in a phase II trial with promising results in prophylaxis [188]. Letermovir (previously known as AIC246) is another anti-CMV agent with a novel mechanism of action targeting the viral terminase subunit pUL56, a component of the terminase complex. This agent demonstrated dose-dependent prophylactic efficacy in a phase II trial [189]. If the ongoing phase III trials confirm these results, a paradigm shift may occur in the future.

New DNA-based vaccination strategies against CMV are being evaluated in clinical trials [190].

Main antiviral recommendations are noted in Table 3.

### Epstein-Barr virus disease prevention

Factors associated with an enhanced risk for Epstein-Barr virus (EBV) replication and therefore infection after allo-HCT are a selective T cell depletion of the graft, a HLA-mismatched transplantation, the choice of an unrelated donor (especially haplo-identical transplant recipients), and the use of T cell depleting antibodies, e.g. alemtuzumab or ATG during conditioning [191, 192]. Early EBV-disease after transplantation is extremely rare. Primary or secondary prophylactic use of antiviral agents is not effective against EBV and therefore not recommended **(DII)** [193, 194]. Close EBV viral load monitoring and rituximab application can be considered as a preemptive therapeutic approach for the prevention of EBV-associated PTLD after allo-HCT in special high-risk patients **(CIII)** [195–197]. Still considered experimental is the application of cytotoxic T cells for the prevention and treatment of Epstein-Barr virus-induced lymphoma in allogeneic transplant recipients [198, 199].

### Toxoplasmosis prophylaxis

Trimethoprim-sulfamethoxazole (TMP-SMX) prophylaxis, administered to most transplant patients to prevent *Pneumocystis jirovecii* pneumonia, is also efficacious in preventing toxoplasmosis disease [200, 201].

Clinical reactivation of toxoplasmosis may occur in the late phase after transplantation in seropositive patients under immunosuppression. However, the risk is considerably low and no primary prophylaxis is recommended **(DIII)** [47, 202, 203]. After a successful therapy of toxoplasmosis, secondary prophylaxis should be administered for at least 3 months **(AII**_**t**_) [204–206] (Table 4).

**Table 4:** Secondary prophylaxis after toxoplasmosis disease

Intention	Intervention	SoR	QoE	Comments	Ref
To prevent relapse of CNS toxoplasmosis	Pyrimethamine (25 mg/day)^a^ + sulfadiazine (orally, 30 mg/kg/d)	A	II_t_	Minimum duration for 3 months, many cases longer	[204, 206]
	Pyrimethamine (25 mg/d)^a^ + clindamycin (intravenously, 1200 mg/d)	B	II_t_		[255–257]
	Atovaquone 750 mg qid	B	II_t_	In patients intolerant to conventional toxoplasmic encephalitis therapies	[206]

^a^Should be combined with folinic acid

### Hepatitis A prevention

The incidence of infections due to hepatitis A varies widely. Prevention of hepatitis A by vaccination of seronegative patients or donors follows general vaccination recommendations. A previous exposure to hepatitis A has no impact on transplant-related complications, thus only serologic testing is recommended. In case of IgM seropositivity of the donor and/or recipient, allo-HCT might be postponed since a high risk of transmission or hepatic complications are associated with acute hepatitis A. Additional prevention of infection of the recipient can be achieved by avoiding potentially contaminated food. If exposed, passive immunization has been discussed controversially even in non-transplant patients.

Following patients’ post allo-HCT, a continuous loss of acquired hepatitis A antibodies has been described over a median time of 48 months, especially in those older than 18 years. Thus, hepatitis A vaccination should be recommended later in adult transplanted patients at risk **(BII**_**t**_**)** [207].

### Hepatitis B prevention (Table 3)

Hepatitis B infection or reactivation contributes to liver-related morbidity and mortality. This is a frequent problem, which occurs in 21–53 % of patients with immunosuppression [208], especially after conditioning regimens containing alemtuzumab [209]. The goal is to avoid impairment of liver function, fulminant liver failure, hepatic sinusoidal obstruction syndrome (SOS), cirrhosis, or even hepatocellular cancer [210].

Preferably, HBsAG-negative donors should be selected. However, if no other HLA-compatible donor is available, a positive donor for transplantation is not absolutely contraindicated **(BIII)**. Although transplantation of HBV-negative patient with stem cells from an infected donor (HBsAG positive) is associated with a high risk of transmission, some patients develop chronic hepatitis B [210]. Donors with active HBV (DNA detection) should receive antiviral treatment, if possible **(AIII)**.

All HBsAG-positive patients awaiting chemotherapy or immunosuppressive therapy should receive antiviral prophylaxis with a nucleoside analogue, regardless of HBV-DNA levels. In anti-HBc positive patients with no detectable viral replication (resolved HBV infection), there is a serious risk (up to 50 %) of reverse seroconversion after allo-HCT [211]. These patients should be monitored for HBV replication on a regular basis and receive preemptive antiviral treatment with lamivudine **(AII)** or entecavir **(AII)** once HBV DNA levels are positive (more details: Table 3). It is recommended that antiviral treatment should be continued until at least 6 months after the cessation of immunosuppression **(BIII)** [29]. Prophylactic treatment of anti-HBc-positive patients without any viral load during the first months after allo-HCT is optional since no data are published in this patient population **(CIII)**.

### Hepatitis C prevention

Patients tested positive for HCV-RNA have a significantly higher risk of developing sinusoidal obstruction syndrome (SOS). Long after allo-HCT they suffer a higher rate of liver fibrosis or cirrhosis [34]. Therefore, patients tested positive for HCV-RNA should be considered (if time permitted) to receive highly active antiviral treatment **(BII**_**t**_**)** [212]. In allo-HCT, data is lacking; however, Mahale et al. reported that patients who have successfully eliminated HCV are not at risk of reactivation at least after conventional chemotherapy [212]. Detailed therapy recommendations cannot be provided since at this time many promising trials with new drugs are being published demonstrating eradication of HCV [213].

Allo-HCT from an HCV-RNA-positive donor should be avoided since the incidence of transmission remains high (**DII**). If timing permitted and no alternative donor options are available, the donor should be treated accordingly to prevent hepatic complications **(AII**_**t**_**)** [214].

### Prevention of diseases caused by respiratory viruses

In recent years, an increasing number of reports on respiratory viral infections after allo-HCT are noted, which are in part attributable to improved diagnostic tools and better awareness. RSV followed by influenza, parainfluenza, metapneumovirus, and adenovirus are the main viruses causing severe diseases [215]. These viruses can contribute significantly to morbidity after allo-HCT; however, mortality rates seem to be mixed due to heterogeneity of various risk situations [216]. The main recommendation is to avoid infections with these viruses through adequate exposure prevention **(AIII)** [217, 218]. Visitors and staff with signs and symptoms of respiratory infections must avoid visiting the wards to prevent further disease **(AIII)**. Additionally, annual influenza vaccination is strongly recommended for healthcare workers, all persons living with allo-HCT candidates or patients to prevent transmission **(AIII)** [219]. If vaccination was carried out during an influenza outbreak, a 2-week course of antiviral chemoprophylaxis could follow until immune response is effective **(BIII)** [19, 220].

There is no published data confirming clinical efficacy of prophylactic administration of respiratory syncytial virus (RSV) immune globulin (RSVIG); therefore, this approach is discouraged **(DII)** [221].

### Intravenous immune globulin for prophylaxis

There is an ongoing controversy about the benefit, dosing, and optimum preparation (hyperimmune or polyvalent) of intravenous immune globulins (IVIG) in allo-HCT [222]. Older studies have demonstrated prevention of infection, interstitial pneumonia (IP), or GVHD [223, 224]. Large meta-analyses demonstrated no clinical benefit, except for a decrease of IP and an increase of sinusoidal obstruction syndrome (SOS) with high-dose IVIG [225, 226].

In a recent multicenter trial, 200 patients received different doses of IVIG or placebo weekly starting day −7 till day +100, but no differences were observed in regards to infections, interstitial pneumonia, treatment-related mortality, and overall survival. However, higher doses of immune globulin were again associated with deleterious SOS [227]. Therefore, the routine prophylactic substitution of immune globulin is not recommended if the IgG level is >4 g/L **(DI)** [227, 228].

Nevertheless, a retrospective study reported patients with severe hypogammaglobulinemia (e.g. IgG <4 g/L) were to be at risk for decreased survival [229]. This compares well with the IgG substitution recommendations by the IDSA [220] and the guidelines for patients with the common variable immunodeficiency (CVID) syndrome to substitute low-dose immune globulin if IgG <4 g/l [230]. According to an analysis by the Cochrane group, the use of IVIG may be considered in patients with hypogammaglobulinemia associated with CLL or multiple myeloma and recurrent infections. IVIG can significantly decrease the number of infections [225, 226].

Therefore, immune globulin should be replaced in patients with low serum IgG levels and recurrent infections associated with hypogammaglobulinemia to lower the incidence of infections **(BII**_**t**_**)**.

### Granulocyte transfusion for prophylaxis

A small matched pair analysis of nine neutropenic patients at high risk for recurrence of a previous fungal infections after allo-HCT demonstrated that prophylactic administration of granulocyte transfusions could reduce the incidence and shorten the duration of fever as well as the duration of neutropenia compared to the control group [231]. Oza et al. performed a “biological randomization” in 151 stem cell recipients dependent on ABO- and CMV-compatibility of their donor. There was a significant decrease in the number of febrile days and the use of intravenous antibiotics; however, no difference in the length of hospital stay or 100-day survival was noted [232]. So far, prophylactic granulocyte transfusion remains an experimental approach and is considered more a therapeutic option.

## Immunization (Tables 5 and 6)

**Table 5:** Vaccination recommendations for allo-HCT recipients

Intention	Intervention (timing of 1st application after allo-HCT)				SoR	QoE	Comments	Ref
Vaccine		after day +100	after 6–12 months	after 24 months				
Provide immunity	*Pneumococcus* (combination of conjugate and polysaccharide vaccines)	X			A	II_t_	Post allo-HCT 6 months; 3 applications of 13-valent pneumococcal conjugate vaccine (PCV13, 4 weeks apart). After 1 year post allo-HCT use 23-valent polysaccharide pneumococcal vaccine; no data for non-myeloablative, haplo-identical, or DLI protocol regimes	[258, 269–270, 310]
	*Pneumococcus* (polysaccharide vaccine) 23-valent (PPV23)		(X)		D	II	Alone not recommended as a single vaccine since conjugate vaccine provide a better immune response	
	Influenza	X			A	II	Consider to vaccinate patient after 4 weeks again (BII) if still early after transplantation; Include next of kin and healthcare workers (HCW) to receive vaccination as well (AIII). Consider quadrivalent vaccine (BIII).	[266, 271–276]
	*Bordatella pertussis* (acellular)^b^		X		A	III	Antibody levels do not reflect effective vaccination.	[276–278]
	Diphtheria and tetanus toxoid^a, b^		X		A	II	First diphtheria and tetanus vaccination after 12 months; only data available beyond 12 months; for diphtheria higher dose possibly better (child dosage) but not approved (BIII)	[279–281, 310]
	TBE^a^ (Tick-borne encephalitis)		X		B	II_t_	Only in endemic areas	[282]
	Poliovirus^a, b^		X		A	II	Inactivated vaccine only	[281, 283–288]
	*Haemophilus influenza* (HI)^a, b^		X		B	II	Incidence of HI type B (vs. non type B) infections after allo-HCT is relatively low.	[269, 288–295, 310]
	Meningococcal conjugate vaccine against serogroups A, C, W135, Y and Meningococcal vaccine for serogroup B		X		B	II_t_	Europe, North America and Canada register high rates of meningococcal disease by serogroup B. Therefore, both vaccines are recommended equally.	[295–297]
	Hepatitis A and B (HAV and HBV)^a, b^		X		B	II_t_	Only in patients at risk for hepatitis. Combination vaccination possible. Patients with a previous history of HBV need to be revaccinated **(AII)**.	[33, 207, 298–300]
	MMR (mumps, measles, and rubella; life attenuated vaccine)^a, b^			X	B	II	Live attenuated vaccine after 24 months post allo-HCT and no GVHD or immunosuppressive therapy. Less than 24 months: DIII. Consider occupational risks and household.	[301–303]
	VZV (varizella zoster virus, life attenuated vaccine) ^a^			X	B	II	As MMR but no history of VZV disease and seronegative	[304–307]

^a^Consider antibody measurements

^b^Consider combination vaccines

**Table 6:** Immunization schedule

Vaccine	SoR/QoE	Relative to day of allo-HCT				12 months after first vaccination	Refresher	Comments	Ref
		Day +100	Month +6	Month +7	Month +8				
Pneumococcus	AII_t_	X	X	X		X	Unclear	Start with PCV13, after 12 months after first vaccination the 23 valent polysaccharide vaccine should be used	[269–270, 308]
Influenza	AII	X				X	Annually		[266, 271–276]
Polio inactivated^a^	AII		X	X	X	X	According to local health advisory	^a^ Combination vaccine possible	[283–288, 309]
Pertussis (acellular)^a^	AIII		X	X	X	X			[277, 278, 309]
Diphtheria and tetanus toxoid^a^	AII		X	X	X	X			[279–282, 292, 309]
*Haemophilus influenzae* ^a^	BII		X	X	X	X			[269, 284, 288–295, 311, 312]
Meningococcal conjugate 4 valent and serogroup B	BII		X			X	None		[295, 313–315]
TBE	BII_t_		X	X		X	5 years		[282, 316]
Hepatitis B	CII		X			X	Depending on titer	Combination vaccine possible (together with HAV)	[298-299, 317–320]
Mumps, measles, rubella	BII	Live attenuated vaccine after 24 months accordingly to the rule mentioned in Table 5					Unclear, depending on immune response	One dose is recommended	[301–303, 321, 322]
Varicella zoster virus (VZV)	BII								[304–307, 323]

^a^combination vaccination possible

Protection against vaccine-preventable infections should be a part of the post-transplantation medical care management. Ideally, trials should provide evidence for the protection against diseases. However, a study powered for protective efficacy is not necessary if a sponsor of a vaccine study can justify the use of immunological data to predict protection against infection [233]. If it is not feasible to perform an efficacy study and there is no immunological correlation of protection, it may sometimes be justifiable to gauge the likely efficacy of a vaccine by comparison of immunological responses with those seen in past studies of similar vaccines with proven protective efficacy (e.g., acellular pertussis vaccines) [233].

In allo-HCT recipients, antibody titers against vaccine-preventable infections decline, leading to an increased risk of developing a disease [259]. For this reason, an early vaccination schedule would be warranted. However, during the first 3 to 6 months after transplantation, a sufficient specific immune system response to vaccination cannot be expected [259]. Depending on different factors such as pre- and post-transplant treatment, age, type of transplantation, or presence of chronic GVHD, recovery of the immune system is delayed [259]. Additionally, limited information about vaccine response exists for patients after reduced-intensity conditioning or with umbilical cord blood grafts. Administration of rituximab can suppress humoral immune response as long as 6 months after the last dose. Delayed vaccination schedules should be considered in these patients **(BII**_**t**_**)** [260–262].

Pneumococcal and meningococcal immunization with the conjugated vaccine seems to provide a more stable immune response than the polysaccharide-based vaccine in immature or altered immune systems, but comparative trials are still missing [263].

MMR (measles, mumps, rubella) and *Varicell*a vaccines are live attenuated vaccines that should not be given within the first 2 years after transplantation or during active GVHD **(DIII)**. A significant risk of disease and side effects in the immunocompromised patient were observed. However, 24 months after transplantation without evidence of chronic GVHD and immunosuppression, MMR vaccine appears safe to be administered **(BIII**).

Routine VZV-vaccination is currently not indicated in seropositive patients for the prevention of herpes zoster **(DIII)**. A newer inactivated VZV vaccine is being developed providing adequate VZV-specific antibody titers in most patients [264]. This new vaccine has the potential to change this recommendation in the future.

Additional immunizations against hepatitis A virus, human papillomavirus, yellow fever, cholera, typhus, rotavirus, or pre-exposure rabies virus vaccination are not routinely indicated in adults. Decision-making should follow the recommendations of general population and country-specific policy. Degree of immune suppression against live attenuated vaccines especially in the allo-HCT population needs special attention.

Little is known whether vaccinations can induce GVHD, since viral infections are known to do so [265]. On the other hand, clinical data demonstrate response to vaccination despite GVHD [266, 267]. A group of European experts published results of a consensus conference on vaccination in GVHD [268]. The conference attendees were more cautious about immune suppression. In patients receiving prednisone ≥0.5 mg/kg bodyweight per day as part of a combination therapy or a three-agent immunosuppressive treatment is given; vaccination may be postponed until immunosuppression is reduced to a double combination or prednisone <0.5 mg/kg bodyweight daily in order to achieve a better vaccine response **(BIII)** [268].

Antibody titer testing prior to and after immunization can be recommended for many vaccines. Decision-making based on a titer is not recommended for all vaccinations to document efficacy except for VZV, HAV, or HBV. Basically, titer determination provides some insight on vaccination success and should be considered as optional **(CIII)**. Testing for sufficient antibody response after immunization is indicated in hepatitis B one month or later after the third vaccine dose **(BIII)**. Revaccination with a second series of hepatitis B vaccine should be considered in non-responders **(CIII)**.

In review of the available literature, clearly more studies are needed to provide more information on the safety and efficacy of vaccination schedules in allo-HCT.
